# Selective zygomaticus muscle activation by ball electrodes in synkinetically reinnervated patients after facial paralysis

**DOI:** 10.3389/fresc.2023.1205154

**Published:** 2023-10-16

**Authors:** Dirk Arnold, Jovanna Thielker, Carsten M. Klingner, Orlando Guntinas-Lichius, Gerd Fabian Volk

**Affiliations:** ^1^ Department of Otorhinolaryngology, Jena University Hospital, Jena, Germany; ^2^ Facial-Nerve-Center Jena, Jena University Hospital, Jena, Germany; ^3^ Department of Neurology, Jena University Hospital, Jena, Germany; ^4^ Center for Rare Diseases, Jena University Hospital, Jena, Germany

**Keywords:** electrical stimulation, surface electrodes, facial paralysis, facial palsy, zygomaticus muscle, muscle atrophy, reinnervation

## Abstract

**Introduction:**

Although many different treatments were developed for facial palsy, only a few therapeutic options are available for facial synkinesis. Electrical stimulation of specific muscles via implants could be useful in restoring facial symmetry in synkinetic patients. A challenge in developing stimulation devices is finding the right stimulation location, type, and amplitude. This work assesses the ability to selectively stimulate the zygomaticus muscle (ZYG) in patients with oral-ocular synkinesis to elicit a visually detectable response of the ipsilateral corner of the mouth (COM), without causing a reaction of the orbicularis oculi muscle (OOM). We aimed to assess how close to the COM the stimulation should be delivered in order to be selective.

**Methods:**

A total of 10 patients (eight females, two males) were enrolled. Facial function was graded according to the Sunnybrook facial grading system. Needle EMG was used to test the activities of the muscles, during volitional and “unintended” movements, and the degree of synkinesis of the ZYG and OOM. Two ball electrodes connected to an external stimulator were placed on the paretic ZYG, as close as possible to the COM.

**Results:**

Independent of the waveform with which the stimulation was presented, a selective ZYG response was observed within 4.5 cm of the horizontal plane and 3 cm of the vertical plane of the COM. When the distance between the electrodes was kept to ≤2 cm, the amplitude necessary to trigger a response ranged between 3 and 6 mA when the stimulation was delivered with triangular pulses and between 2.5 and 3.5 mA for rectangular pulses. The required amplitude did not seem to be dependent on the applied phase duration (PD), as long as the PD was ≥5 ms.

**Conclusion:**

Our results show that selective stimulation of the ZYG presenting synkinetic ZYG–OOM reinnervation can be achieved using a broad PD range (25–1,000 ms) and an average amplitude ≤6 mA, which may be further decreased to 3.5 mA if the stimulation is delivered via rectangular rather than triangular waves. The most comfortable and effective results were observed with PDs between 50 and 250 ms, suggesting that this range should be selected in future studies.

**Clinical Trial Registration:**

[https://drks.de/search/de/trial/DRKS00019992], identifier (DRKS00019992).

## Introduction

Facial palsy, which is an umbrella term for incomplete facial paresis and complete facial paralysis, results from temporary or permanent dysfunction of the peripheral facial nerve (FN). Ordinarily, the FN splits into the temporal, zygomatic, buccal, marginal mandibular, and cervical branches (1), which innervate the main facial muscles responsible for mimic and expression. Despite the frequently observed simplification in anatomical literature, the anatomical position of the FN and the path of its various branches have high inter- and intraindividual variability (2–4). This can result in a higher number of distal branches than those commonly described and an increased occurrence of nerve anastomoses and plexus. For example, the innervation of the zygomaticus muscle (ZYG) and the orbicularis oculi muscle (OOM) has been traced back to either the deep buccal or the zygomatic branches (5, 6). Apparently, the distribution of innervating nerve branches is not consistently determined developmentally in these areas. The distribution is influenced to a greater extent by molecules within the extracellular matrix, which causes erratic reinnervation patterns. This finally leads to the development of facial synkinesis (7–9). Facial synkinesis is the most common long-term sequelae of facial paralysis with axonal damage. It is defined as the “unintended” movement of a muscle during the volitional movement of another muscle, through misdirected regrowth of axons during reinnervation after a lesion on the FN (10, 11). The severity of synkinetic reinnervation mainly depends on the size and location of the damage. Since axons have been found to regenerate with an average rate of 1 mm per day (7, 12), clinical signs of synkinesis are usually only detected between 3 and 4 months and 2 years after the nerve injury (10). Facial muscle synkinesis usually includes oral-ocular synkinesis, i.e., unintended eye closure during volitional mouth movement, and ocular-oral synkinesis, i.e., unintended mouth movement during volitional eye closure (13). These can appear alone or in combination with each other. The main problem for patients suffering from facial synkinesis beyond visual disturbances is the inability to properly convey emotions. This in turn can lead to psychological issues, such as depression, social isolation, and anxiety (14–18). Consequently, facial synkinesis often has a strong negative impact on the quality of life of a patient (19–21).

The repertoire of treatment options for facial palsy in general is much more extensive than that for patients with facial synkinesis. Thus, in addition to various training methods (22–27) and surgical interventions to reconstruct the innervation (28, 29), electrical stimulation is deemed as a safe and effective tool for the treatment of facial palsy (30) and in decelerating the atrophy of the affected muscles without negatively affecting muscle reinnervation (31–33).

In contrast, the most common treatment of facial synkinesis is the injection of botulinum toxin into the affected muscle(s) every 3–6 months (34, 35). However, many studies recommend training therapy in addition to or as an alternative to botulinum toxin treatment (36, 37) and in combination with EMG biofeedback (38–40).

Electrical stimulation to overcome the synkinetic activity of individual muscles has lately emerged as a promising approach (41). Based on different studies (42–44), the effectiveness of electrical stimulation is primarily dependent on the ability to activate a specific muscle with appropriate parameters.

Active implants could be a new approach to restoring smile symmetry or eye closure for reinnervated yet facially paralyzed patients. In most synkinetically reinnervated patients, volitional maneuvers such as smiling or blinking are possible to a certain extent, but when an individual tension threshold is exceeded, the synkinetically reinnervated muscles are coactivated. With an implant that detects the activity levels of the target muscles (e.g., left and right ZYG), electrical stimulation could be used to enhance the contraction of the concerned muscle before that threshold, without the patient having to actively increase the activity. Thus, it would be possible to reduce or even avoid the synkinetic effects with selective activation. To date, such implants have only been described in theory (45, 46), and have not been used in clinical practice. Rapid developments in chip-, electrode-, and transmission technology and deep learning approaches provide the perspective that such implants will become possible in the future. However, several problems must first be addressed; for instance, the electrodes should be as thin as a sheet of paper. Recent developments are already moving in the right direction [e.g., tattoo electrodes (47–49)]. In addition, they should be capable of sending electromyography (EMG) signals wirelessly from the healthy side as a trigger, to the control unit on the paralyzed side. A determination of the position at which the stimulation should be placed is therefore imperative (4, 42) because selective stimulation can only be successful if the innervating nerve of the target muscle is stimulated distal to the branching axons that are responsible for the unintended activity. In our opinion, the implantation of a stimulation device would be useful especially when improvements can no longer be achieved with non-invasive methods. However, non-invasive training methods using surface electrodes could also benefit from our results.

The work presented herein assessed the ability to selectively stimulate the ZYG in patients with oral-ocular synkinesis, as diagnosed via needle EMG, to elicit a visually detectable response of the ipsilateral corner of the mouth (COM), without causing a reaction of the ipsilateral eye. In particular, we aimed to assess how close to the respective COM the stimulation should be delivered on the ipsilateral ZYG, to be selective without causing OOM activation. The clear definition of the optimal stimulation region is a critical element of the subsequent development of implantable solutions for the treatment of facial synkinesis.

## Methods

Data were collected from June 2018 to February 2021 at the Facial Nerve Center of the ENT Department, Jena University Hospital, as a longitudinal, open-label, prospective, case series-based, proof-of-principle clinical investigation. This study was approved by the Ethics Committee of Jena University Hospital in 2018 (application number 5403–02/18) and has been registered in the German Clinical Trials Register (Deutsches Register Klinischer Studien, DRKS 00019992). All participants gave written informed consent prior to their inclusion in this study.

### Inclusion and exclusion criteria

The inclusion criteria were as follows: age ≥18 years old; onset of the palsy ≥6 months ago; diagnosed facial palsy with aberrant reinnervation of the ZYG and/or the OOM; anatomic, physiological, and mental conditions compatible with participation; and high motivation with realistic expectations regarding the participation.

The exclusion criteria were as follows: pregnant or breast-feeding women; any head (e.g., facial and/or neck) surgery (except patient 8), pharmacological treatment (e.g., botulinum toxin injection, wrinkle treatment), or physiotherapeutic program within the last 3 months before enrollment; other clinical diseases that might result in alteration of the outcomes (e.g., muscular and/or skin diseases, epilepsy); use of an active medical implant; known allergies or intolerance to the material used for this clinical investigation; ongoing participation in other drug and/or medical device clinical investigations that could affect the results of the present clinical investigation; and in the opinion of the principal investigator, anything that would place the subject at increased risk or preclude the subject's full compliance with the general requirements.

### Patients demographics

A total of 10 patients (eight females, two males) were enrolled in the study. The patients were referred to the ENT Department at Jena University Hospital after already being diagnosed with facial palsy by MRI and clinical examinations in other specialized clinics. The mean age at enrollment was 52.4 ± 11.5 years (range 35–69; median = 51). The interval from the onset of unilateral facial paralysis to the time of enrollment ranged from 6 months to 36 years (Table 1). One patient underwent a reconstructive surgical treatment, 20% of the patients were treated with botulinum toxin (more than 3 months earlier than enrollment), 10% required artificial tears, and 80% underwent speech therapy and/or other physiotherapeutic treatment.

**Table 1:** Demographic characteristics of the patients assessed.

Patient (*n* = 10)	Age	Gender	FP etiology	Side	FP onset	Interval FP onset/ enrollment
1	49	Female	Iatrogenic, revision of parotic tumor	Right	September 2016	1 y 11 m
2	47	Female	Idiopathic	Left	July 2017	1 y
3	38	Female	Herpes zoster	Left	July 1989	29 y 5 m
4	69	Female	Herpes zoster	Right	January 2018	6 m
5	52	Male	Idiopathic	Right	January 2018	6 m
6	35	Female	Iatrogenic, resection of vestibular schwannoma	Left	December 2015	2 y 8 m
7	55	Female	Iatrogenic, resection of vestibular schwannoma	Right	August 2017	1 y
8	69	Female	Iatrogenic, resection of vestibular schwannoma, facial nerve reconstruction by hypoglossal-facial jump nerve suture	Right	September 2011, facial nerve reconstruction, April 2012	7 y 3 m
9	49	Female	Idiopathic	Left	July 1983	35 y 11 m
10	61	Male	Idiopathic	Right	February 2020	12 m

FP, facial paralysis; y, year(s); m, months.

### Procedure and stimulation parameters

Before starting the stimulation procedure, two independent examiners assessed the symptoms of the patients according to the Sunnybrook facial grading system (50). Here, the examiners visually recorded the symmetry at rest, the symmetry during defined movements (raising eyebrows, gently closing the eyelid, smiling with open mouth, showing teeth, pursing lips), and the synkinesis during the same movements. Afterward, they weighted them (symmetry at rest ×5, symmetry at movement ×4, synkinesis ×1) to combine into a total score.

In addition, using needle EMG (Synergy T5, Viasys, CareFusion, San Diego, CA, USA), the examiners evaluated pathological spontaneous activity (PSA) and rated it 0 = none or 1 = present in multiple areas, muscle reactions during the volitional maneuvers of smiling (ZYG) and blinking (OOM), and unintended activity of these muscles during maneuvers in which they would not normally be expected to be activated [blinking (ZYG) and smiling (OOM)] (51). The activity was graded subjectively by the examiner as “none,” “single fiber pattern,” “strongly decreased,” “moderately decreased,” “mildly decreased,” or “normal.” A classification of synkinesis into “none,” “mild,” “moderate,” or “strong” was based on the detected unintended activities of both muscles. Synkinesis was graded as “mild” if the unintended activity of the ZYG or the OOM or both muscles was assessed as “strongly decreased.” In contrast, synkinesis was “strong” if the activity was “mildly decreased” and “moderate” if a moderate attenuation was detected.

**Figure 1:**
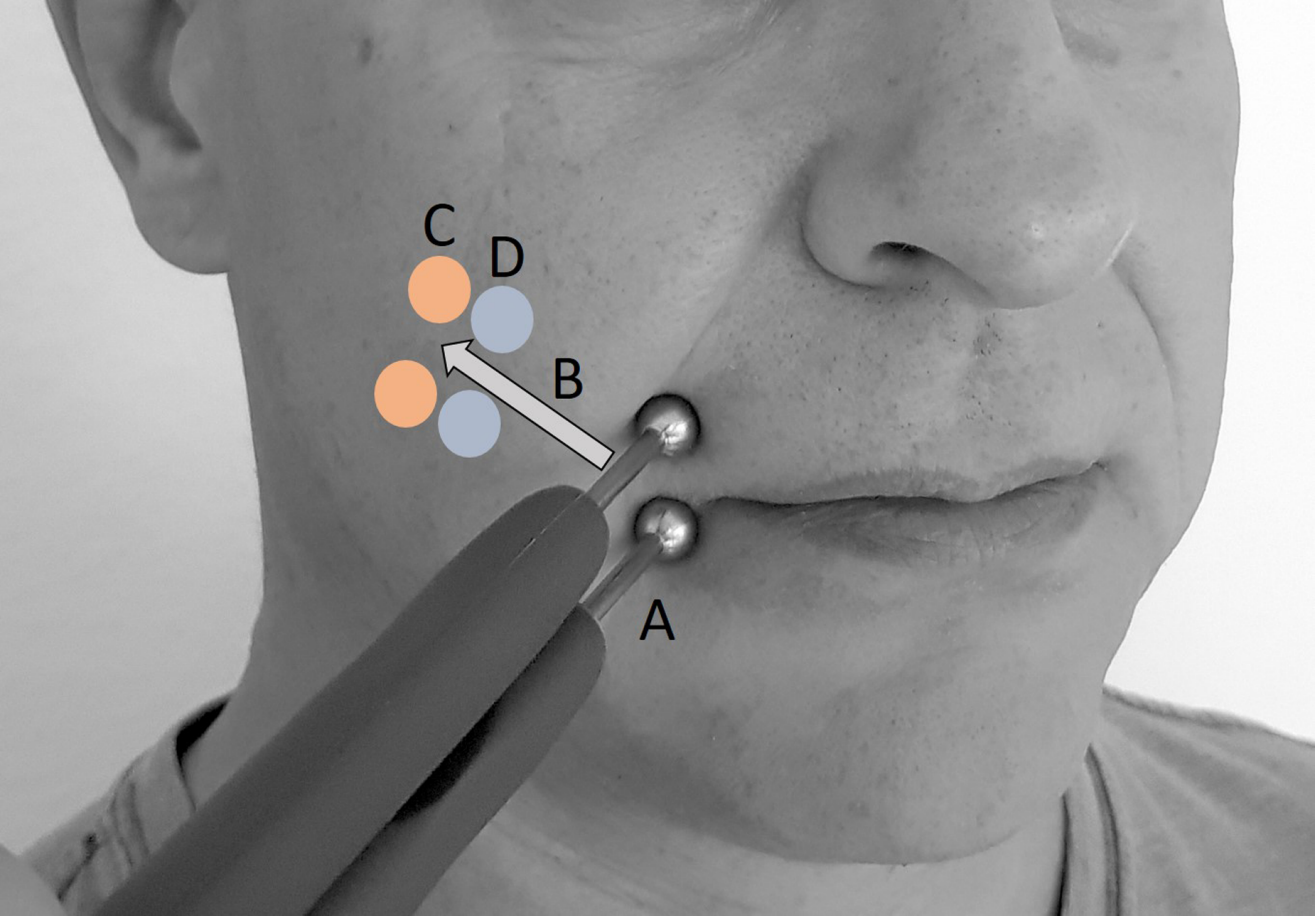
Stimulation procedure with two ball electrodes (conducting ultrasound gel not shown). (A) Starting position for test stimulation. (B) Both electrodes were moved retrograde along the assumed facial nerve path, without detaching either electrode. (C) Position of first unspecific muscle reactions. (D) Last position of specific zygomaticus muscle (ZYG) response and position for all PD tests.

With the patient in a sitting position, two ball electrodes (8 mm Ø; Physiomed Elektromedizin AG, Schnaittach, Germany) connected to an external stimulator (STMIsola, BIOPAC Systems, Inc., Germany) were placed on the paretic ZYG [the skin was prepared using conducting ultrasound gel (not shown in figure) (Sonosid/Asid Bonz GmbH], as close as possible to the respective COM with an interelectrode distance of 1.5 cm (Figure 1). In addition to the research stimulation device used in this study (STMIsola, BIOPAC Systems, Inc. Germany), we have good experience with commercially available stimulation devices for patients that output pulse durations and pulse forms such as those used in this study, e.g., MED-EL's STIWELL, Krauth + Timmermann’s Paresestim, and Schuhfried's Stimulette.

Using biphasic electrical stimulation, delivered with triangular or rectangular waveforms, a phase duration (PD) of 100 ms, and a frequency of 1 Hz, the examiner increased the amplitude from 0.5 to a maximum of 25 mA, in 0.5 mA increments, until a selective ZYG response was achieved without patient discomfort. The stimulation was aimed at the (re)innervating nerve branches. A current-controlled stimulation was used to ensure the consistency of the physiological responses between sessions. The voltage is dependent on the impedance between the electrodes. A selective ZYG response was determined visually as the respective COM lifting. At this point, the ball electrodes were moved retrograde along the assumed facial nerve path, in the direction of the ear, without detaching either electrode from the skin of the patient until a selective ZYG response was no longer observed within the aforementioned amplitude range. The last position where a selective ZYG response was possible without the unintended activity of the OOM or unspecific cocontractions of other muscles and without discomfort being experienced by the patient was used for the analysis. At this point of stimulation, the response of the ZYG to the use of a PD of 1,000, 500, 250, 100, 50, 25, 15, 10, 5, and 1 ms was assessed in this sequence. The whole stimulation procedure lasted up to 25 min per patient.

### Statistical analysis

Data were analyzed using IBM SPSS statistics software (Version 25, IBM, NY, USA) for medical statistics. The distribution of continuous data was described using mean values ± standard deviation. Qualitative data were presented in absolute and relative frequencies. Due to the small sample size, as a proof of principle clinical investigation, no statistical analyses could be carried out and the data were evaluated descriptively.

## Results

### Electromyography assessment

Table 2 shows the results of the EMG examinations for each patient. PSA at multiple areas of the affected ZYG could be detected in only one patient (2). No PSA was observed in any patients in the OOM. All patients were capable of intentional movement of the ZYG to varying degrees. “Normal” activity (09), “strongly decreased” activity (07), and “single fiber pattern” activity (06) were observed once each. The volitional activity was “mildly decreased” in three patients (1, 4, and 5) and “moderately decreased” in four patients (2, 3, 8, and 10). In contrast, no volitional activity of the OOM was observed in two patients (7 and 8). A “strongly decreased” volitional activity was observed in one patient (4) and “mildly decreased” volitional activity also in one patient (1). Four of the patients (2, 3, 6, and 10) had a “moderately decreased” volitional OOM activity, and two patients (5 and 9) had “normal” OOM activity.

**Table 2:** Needle electromyography results of all patients during volitional maneuvers such as smiling (ZYG) or blinking (OOM) or unintended maneuvers such as blinking (ZYG) or smiling (OOM).

Patient (*n* = 10)	PSA		Volitional activity		Unintended activity		Synkinesis
	0	1	ZYG	OOM	ZYG	OOM	
1	OOM ZYG		Mildly decreased	Mildly decreased	Moderately decreased	Moderately decreased	Moderate
2	OOM	ZYG	Moderately decreased	Moderately decreased	Mildly decreased	Mildly decreased	Strong
3	OOM ZYG	** **	Moderately decreased	Moderately decreased	Moderately decreased	Moderately decreased	Moderate
4	OOM ZYG	** **	Mildly decreased	Strongly decreased	Moderately decreased	Strongly decreased	Mild
5	OOM ZYG		Mildly decreased	Normal	Moderately decreased	Moderately decreased	Moderate
6	OOM ZYG		Single fiber pattern	Moderately decreased	Strongly decreased	Moderately decreased	Mild
7	OOM ZYG		Strongly decreased	None	Strongly decreased	Single fiber pattern	Mild
8	OOM ZYG		Moderately decreased	ND	ND	Single fiber pattern	Mild
9	OOM ZYG		Normal	Normal	Moderately decreased	Moderately decreased	Moderate
10	OOM ZYG		Moderately decreased	Moderately decreased	Moderately decreased	Moderately decreased	Moderate

PSA, pathological spontaneous activity; ZYG, zygomaticus muscle; OOM, orbicularis oculi muscle; ND, not determined.

Finally, the following synkinesis classifications resulted from the measured unintended muscle movements: The ZYG–OOM synkinesis was “strong” in only one patient (2), “moderate” in five patients (1, 3, 5, 9, and 10), and “mild” in four patients (4, 6, 7, and 8).

### Sunnybrook facial grading system assessment

The mean composite score was 45.9 ± 22.4 (range 5–80; median = 47.5), representing a “moderate” post-paralytic facial syndrome. A moderate loss of resting symmetry was confirmed by a mean resting symmetry score of 10 ± 4.1 (range 5–15; median = 10). The average voluntary movement score was 61.2 ± 22.3 (range 20–88; median = 64). This corresponds to a “moderate” impairment of volitional movement. There was a “mild”-to-“moderate” synkinesis between the ZYG and the OOM with a mean synkinesis score of 5.3 ± 3.4 (range 0–10; median = 6).

### Safety

No adverse events were observed for the entire duration of the study.

### Selective zygomaticus muscle response

Independent of the waveform of the stimulation, a selective ZYG response was only observed with stimulation within approximately 4.5 cm of the horizontal plane and 3 cm of the vertical plane from the respective COM. It was not possible to determine a discomfort threshold that was valid for all the patients, because the subjective sensation varied greatly between the patients. The patients who had already undergone electrical stimulation tended to show greater sensitivity thresholds than patients for whom it was the first electrical stimulation.

**Figure 2:**
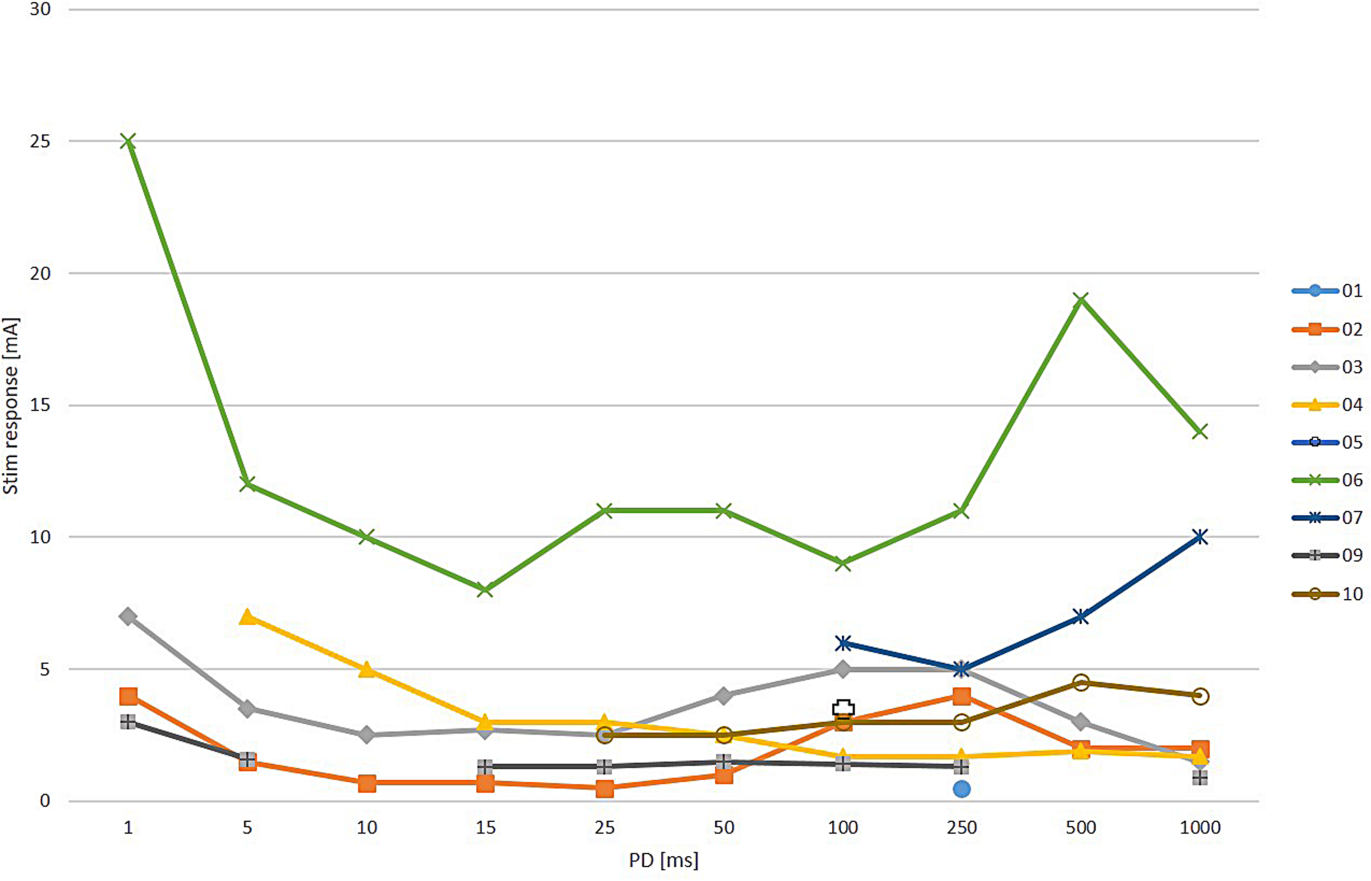
Results of zygomaticus muscle (ZYG) stimulation presented as triangular waves at various phase durations (PDs in ms). No stimulation response (Stim response) at any amplitude (mA) as selective ZYG activation was possible in patient 8 for any tested PD, because the threshold of discomfort was always reached before. For patients 1 and 5, a selective ZYG response could be elicited only with 100 and 250 ms, respectively. Interruptions in the lines within the line chart occur when the ZYG of a patient does not respond to each PD with a selective activity.

When wave stimulation with triangular pulses was used, a selective ZYG response was observed in this area in nine patients (Figure 2). In patient 8, the threshold of discomfort was reached before the ZYG could be activated selectively with PDs between 15 and 1,000 ms. Shorter PDs were not tested for that reason. Among the responders, in patient 5 a specific ZYG response could only be observed with 100 ms, while discomfort threshold was reached with the other PDs tested (250, 500 and 1000). Only 250 ms was effective in patient 1. The other PDs were not completely tested because they caused discomfort and/or unspecific responses to the patient. A mean amplitude of 4 ± 2.5 mA (range 1.4–9; median = 3.3 mA) was required to elicit a selective ZYG response with a PD of 100 ms.

**Figure 3:**
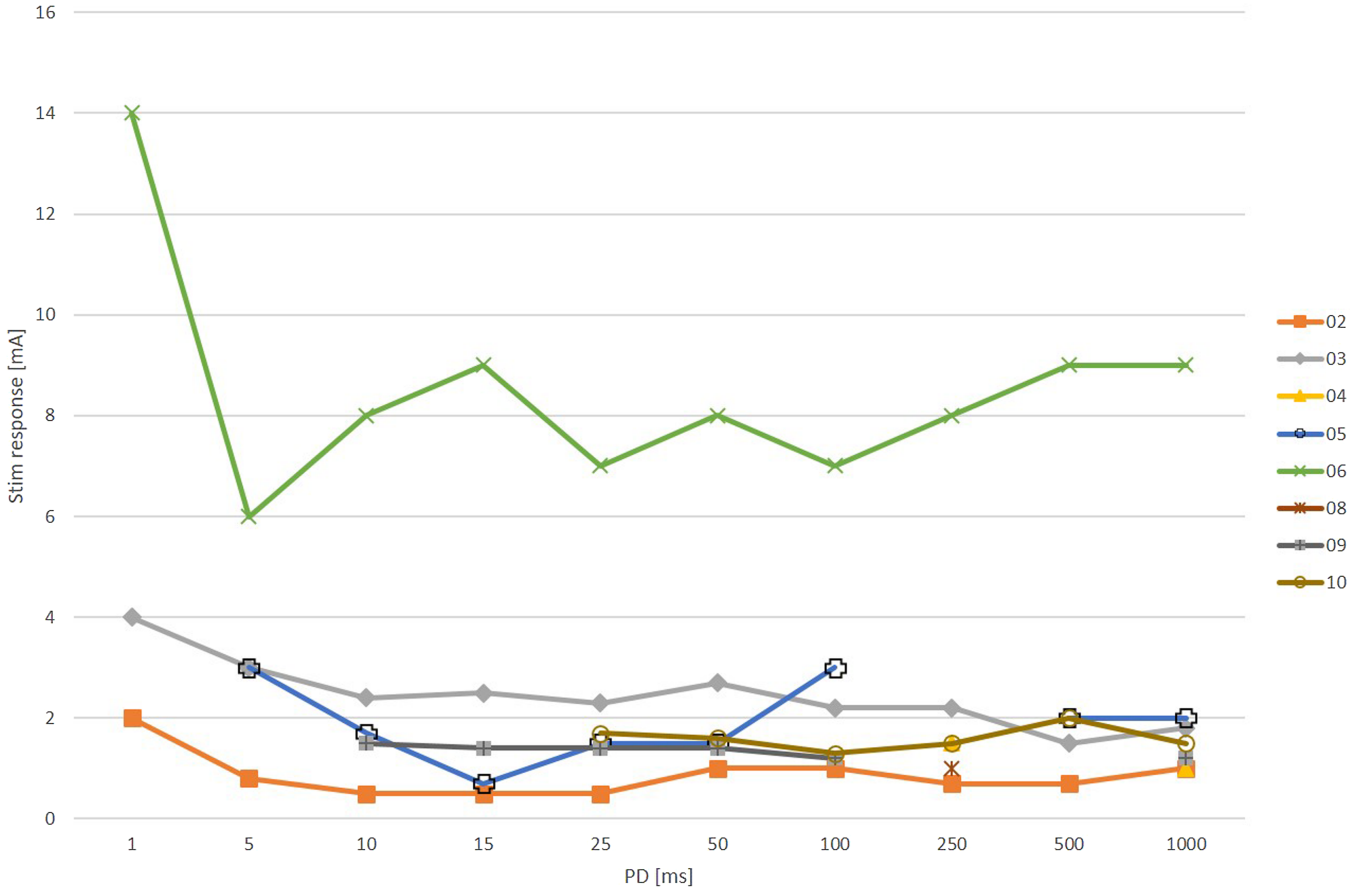
Results of zygomaticus muscle (ZYG) stimulation presented as rectangular waves at various phase durations (PDs in ms). Patient 1 declined to perform the test and only unspecific facial muscle reactions were detectable in patient 7 (data not shown). A selective ZYG activation was possible in patient 8 only with a PD of 250 ms and PDs of with 250 and 1,000 ms in patient 4. As mentioned before, interruptions in the lines occur when the ZYG of a patient does not respond to each PD with a selective activity (Stim response = stimulation response amplitude in mA).

Stimulations with rectangular pulses were performed in nine patients because one patient (1) declined to perform the test. Stimulation successfully triggered a selective ZYG response in eight of the nine patients. In one patient (7), only unspecific reactions without discomfort were observed with a PD range of between 50 and 1,000 ms (data not shown), and other PDs were not tested. The remaining eight patients showed selective stimulations with different PDs, but patient 8 only with 250 ms and patient 4 with 250 and 1,000 ms. Other PDs caused either discomfort or unspecific facial muscle response. A mean amplitude of 2.6 ± 2.3 mA (range 1–7; median = 1.8 mA) was required to observe a selective ZYG response with a PD of 100 ms. The results of all patients (*n* = 10) and all PDs are shown in Figure 3.

The numbers of successful selective ZYG responses for all PDs of triangular and rectangular waveforms are presented in Table 3. Again, patient 1 declined the test. The PDs of 100 ms and 250 ms were most effective for triangular pulses and 1,000 ms for rectangular pulses.

**Table 3:** Number and percentage of successful stimulations of the zygomaticus muscle with different phase durations and waveforms*.*

Waveform	Phase duration (ms)									
	1	5	10	15	25	50	100	250	500	1,000
Triangular	4/10	5/10	4/10	5/10	6/10	6/10	8/10	8/10	6/10	7/10
%	40	50	40	50	60	60	80	80	60	70
Rectangular	3/9	4/9	5/9	5/9	6/9	6/9	6/9	6/9	5/9	7/9
%	33	44	56	56	67	67	67	67	56	78

**Figure 4:**
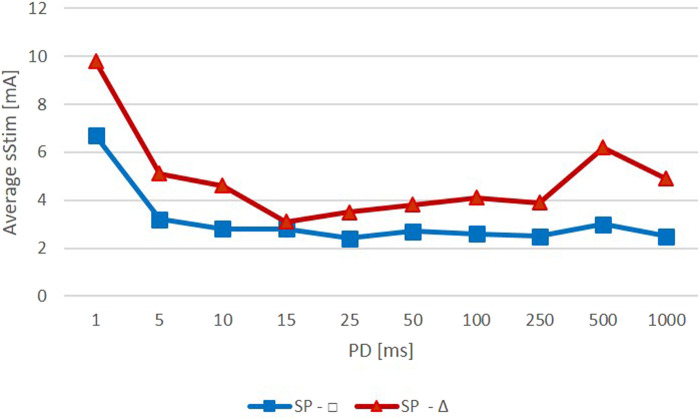
Average amplitude required to observe a specific zygomaticus muscle (ZYG) response with stimulation delivered as triangular (red) or rectangular (blue) waveform (Average Stim, average surface stimulation in mA; SP, stimulation pulse; PD, phase duration in ms).

The mean threshold amplitudes of the rectangular pulses were slightly lower and did not vary as much as those of the triangular pulses (Figure 4). The mean amplitudes for rectangular pulses were all around 3 mA except for a PD of 1 ms, which required an amplitude of approximately 7 mA. Stimulations with triangular pulses required amplitudes between 3 and 5 mA except for the PDs of 1 ms and 500 ms, which required amplitudes of 10 mA and 6 mA, respectively.

## Discussion

The EMG assessment showed that, prior to the testing session, four patients (40%) had a “mild,” five patients (50%) had a “moderate,” and one patient (10%) had a “strong” ZYG–OOM synkinesis. The Sunnybrook assessment confirmed these results and showed that all the enrolled patients had a “moderate” impairment of volitional mouth and eye movements and had a “moderate” loss of facial symmetry (52). The composite score results confirmed “moderate” facial paresis. While both assessments provided comparable results concerning facial paresis symptoms and the presence of synkinetic reinnervation, EMG was found to be more sensitive in detecting initial, subclinical signs of reinnervation (e.g., single fiber pattern) before its effect became clinically relevant and thus assessable through visual outcome measures, such as the Sunnybrook assessment.

The wide range of inter- and intraindividual variation of the innervation path of the FN is well-known, (1–6, 53–57). Similarly, the pattern of synkinetic reinnervation can also be highly variable as a consequence (58, 59). The EMG data presented supports this. In all patients studied, the ZYG and OOM were activated to some extent when smiling or blinking was performed and their activation should not have occurred.

The irregularity of the distribution of the sprouting nerve endings of different motor units during reinnervation and the extent of their re-connection to an undetermined number of muscle fibers in one or more muscles determine the degree of synkinesis (60). The axons may be concentrated in a particular region or may be more widely distributed throughout the muscle. Therefore, when considering the best position for the placement of subcutaneous implantable electrodes for selective stimulation of the ZYG, to restore at least partial facial symmetry, it is important to understand which area of the cheek should be targeted. We were able to observe a selective ZYG response to electrical stimulation delivered, with either triangular or rectangular waveforms, as long as it was delivered on the relevant branch of the facial nerve within an area of approximately 4.5 cm × 3.0 cm above the respective COM. This area was not that different between patients, which indicates that at least in the 10 patients included in this study, the synkinetic axons branch proximal to this area.

In patients with synkinetic reinnervated facial muscles, the stimulation parameter selection is crucial to avoid synkinetic responses or discomfort. PD and amplitude determine the size and strength of the electrical field and, thus, which nerve branches, or even denervated muscle fibers, were activated by the pulses. The distance between the electrodes is also important to create a selective electrical field. If the distance was kept at ≤2 cm, as in the present study, the amplitude necessary to trigger a selective ZYG response would be in a range of between 3 and 6 mA or between 2 and 3 mA, depending on whether the stimulation was delivered with a triangular or a rectangular waveform, respectively. The amplitude required did not seem to depend on the PD applied as long as the latter was ≥5 ms. For lower PDs (e.g., 1 ms), an amplitude equal to twice that value was required to see the same effects with longer PDs. This observation was expected, because short PDs require higher amplitudes to reach the same effect as longer ones (≥5 ms), due to the very short delivery time and the reduced electrical field that is generated, which causes poorer nerve and muscle fiber recruitment and activation (61).

A relevant difference between stimulation of paralyzed vs. post-paralyzed ZYG is that in the former a selective response is achieved with a combination of PD and amplitude that are inversely proportional (61); a shorter PD requires a higher amplitude. In contrast, in the case of post paralyzed ZYG, the amplitude did not change with increasing PDs ≥5 ms. This difference is the result of the presence of reinnervated fibers in the post-paralyzed muscles that are ready to respond to stimulations with short PDs and low amplitudes, which are absent in the paralyzed muscles. Accordingly, in paretic muscles, even a low-magnitude electric field would be able to activate a sufficient number of intact nerve fibers/motor units to elicit a visually detectable response. Furthermore, the plateau beyond which no further intensity increase would improve the ZYG response is more easily reached in post-paralyzed than in completely denervated paralyzed muscles. Still, simultaneous activation of denervated muscle fibers cannot be ruled out since, as recently shown by Arnold et al. (42), they are particularly reactive to longer PDs and lower amplitudes. However, the article cited 6 cm × 4 cm hydrogel electrodes were used, which are much bigger than the ball electrodes used in the present study and produce a larger electrical field with each PD (43).

The PD of 500 ms stimulated with triangular pulses was an exception that required higher amplitudes (in addition to the 1 ms PD). A slightly greater amplitude, compared with the amplitudes at the other PDs, was required to elicit a selective ZYG response (Figure 4). However, this was due to the amplitude value of patient 6 (500 ms, 19 mA) (Figure 2). In this patient, higher amplitudes for all PDs were required to activate the ZYG. That is the only patient in which “single fiber patterns” were detected when testing the volitional activity of the ZYG, i.e., a very weak reinnervation of the muscle was present. Also, the unintended activity of the muscle was “strongly decreased.” This means that in this patient, only a few muscle fibers could be reached via intact nerve branches by electrical stimulation and consequently a greater current was required to elicit a visible response from the ZYG.

Similar EMG results were observed in patient 7. Again, the volitional and unintended activity of the ZYG was “strongly decreased,” and the muscle could only be visibly activated in response to stimulation with longer PDs (100–1,000 ms) and slightly increased amplitudes (Figure 2). Using rectangular pulses, selective activation of the ZYG was not possible at all. There were always unspecific muscle responses in the face before the threshold was reached, which could be explained by the increased electrical field.

As the present study was primarily aimed at the selective response of the ZYG, the strength of its volitional and unintentional activation certainly plays a decisive role in the search for the required amplitude. Similar to the optimal stimulation parameters, a suitable electrode position is highly patient-specific, and their determination is important for adequate performance of electrical stimulation in patients with synkinesis after facial nerve palsy. At present, it is relevant for electrical stimulation with surface electrodes, especially for practicing the denervated or reinnervated muscles, and we could show that selective activation is also possible in synkinetically reinnervated muscles. However, in the future, when the implantation of electrodes is established, it might be also important for implants. This will also depend on the kind of electrode systems, which will be used (intraneural electrodes, extraneural cuff electrodes, or electrodes embedded into muscle tissue). As already mentioned, stimulations delivered as triangular waves released less energy than those delivered as rectangular waves. This is an important observation for the future development of implantable electrical stimulation devices that could help to optimize the lifetime of an implant. Further considerations are that since the implanted electrodes would deliver the stimulation directly on the target nerve/muscles, thus bypassing the connective tissue and the skin, they would most likely be compatible with lower amplitudes than those used in this study. Finally, it is important to note that none of the patients enrolled in this study suffered any adverse events during or after the conclusion of the stimulation session, which confirms the high safety profile of surface electrical stimulation, even when used on very sensitive body regions, such as the face.

## Limitations

The main limitation of our study was the subjective assessment of volitional and unintended activities by the examiners. The evaluation of the degree of synkinesis was performed by facial palsy experts supported by needle EMG to objectify the assessments. Demeco et al. (62) developed a grading system that can be used in future studies to further enhance the objectification of the findings. Büchern et al. (63) also developed another approach to objectify and standardize facial analysis using 3D sensors.

Moreover, our sample size was small. The intention was a proof of principle clinical investigation. This has been successfully achieved. Next step, the results have to be confirmed in a larger sample also allowing detailed statistical analyses. Because we did not permanently treat the patients with the described parameters and we have not investigated the stimulation effects on nerves, musculature, or connective tissue, it is not possible to recommend treatments for rehabilitation or training with specific stimulation parameters based on this publication.

## Conclusions

Surface electrical stimulation can be used to activate specific facial muscles safely and selectively without a synkinetically reinnervated patient's discomfort and/or unselective activation of other ipsi- and/or contralateral muscles. In particular, the selective stimulation of the ZYG showing synkinetic ZYG–OOM reinnervation can be achieved using a broad range of PD (25–1,000 ms) and an average amplitude ≤6 mA that may be decreased further to 3.5 mA if the stimulation is delivered via rectangular rather than triangular waves. The most comfortable and effective results were observed with PDs between 50 and 250 ms, suggesting that this range should be preferred in future studies. We observed that the electrode position on the face and the distance and configuration are critical to observe a specific response within the observed range.

In summary, our results showed that specific stimulation of at least some facial muscles, such as the ZYG, would be compatible with an implantable solution that would represent an interesting alternative for patients suffering from facial synkinesis and not profiting from the currently available standard treatments.

## Data Availability

The original contributions presented in the study are included in the article/Supplementary Material; further inquiries can be directed to the corresponding author.
